# Characterization of the complete chloroplast genome of *Liparis gigantea* (Orchidaceae)

**DOI:** 10.1080/23802359.2024.2415134

**Published:** 2024-10-16

**Authors:** Xingyou Jiang, Wenting Yang, Caixia Peng, Kunlin Wu, Lin Fang, Jingjue Zeng, Songjun Zeng, Lin Li

**Affiliations:** a Key Laboratory of South China Agricultural Plant Molecular Analysis and Genetic Improvement, South China Botanical Garden, Chinese Academy of Sciences, Guangzhou, Guangdong, China; b Guangdong Provincial Key Laboratory of Applied Botany, South China Botanical Garden, Chinese Academy of Sciences, Guangzhou, Guangdong, China; c University of Chinese Academy of Sciences, Beijing, China

**Keywords:** Chloroplast genome, Orchidaceae, phylogeny, *Liparis gigantea*

## Abstract

*Liparis gigantea* is a Chinese traditional medicinal herb in the Orchidaceae family. It is a rare and special *Liparis* species that exhibits relatively large flowers. To illuminate its phylogenetic status and augment genomic resources, the complete chloroplast (cp) genome of *L. gigantea* was first sequenced and assembled using whole genome next-generation sequencing in this study. The cp genome size is 158,462 bp with a total GC content of 36.9%. Characterized by a quadripartite structure, the genome consists of a large single-copy (LSC) region of 86,032 bp, a small single-copy (SSC) region of 18,322 bp, which is separated by a pair of 27,054 bp inverted repeat regions (IRs). A total of 133 genes were annotated, including 87 protein-coding genes, 38 tRNA genes, and 8 rRNA genes. Phylogenetic analysis strongly supported *L. gigantea* as the sister to two closely related terrestrial species, *Liparis nervosa* and *L. vivipara*. The results of this study provide genomic information for future research and application of this medicinal herb.

## Introduction

*Liparis gigantea* C.L. Tso 1933, commonly referred to as Zi Hua Yang Er Suan (purple-flowered sheep ear garlic) by the locals, is a member of the genus *Liparis* Rich. (Epidendroideae, Malaxideae, Malaxidinae). It is a terrestrial, occasionally lithophytic orchid species with plicate leaves that is distributed in southern and southwestern parts of mainland China (e.g. Guangdong, Guangxi, Hainan, Fujian, Yunnan, Guizhou, and Xizang), Hong Kong and Taiwan, also occurring in Thailand, Vietnam, and Northeast India (Zhan et al. 1999; Chen et al. 2009; Wang 2014; Yang et al. 2021). This herbaceous plant has large, dark purple flowers, and naturally grows in shaded and damp places of evergreen broad-leaved forests, at altitudes ranging from 500 to 1700 m (Chen et al. 2009). Like most other species of this genus, *L. gigantea* has long been used as a traditional Chinese herb medicine for its removing dampness and detoxicating properties, as well as effectiveness in rheumatic arthralgia, dermatitis, bruises, and swelling sores (Liang et al. 2019).

Chloroplast genomes have greatly improved the phylogenetic resolution at all taxonomic levels in angiosperms (Yang et al. 2022; Chen et al. 2023; Yang et al. 2023). To clarify the phylogenetic position of *L. gigantea* and to better understand this beneficial plant, the complete cp genome of *L. gigantea* was first assembled and characterized using the DNBSEQ platform.

## Materials and methods

**Figure 1.**
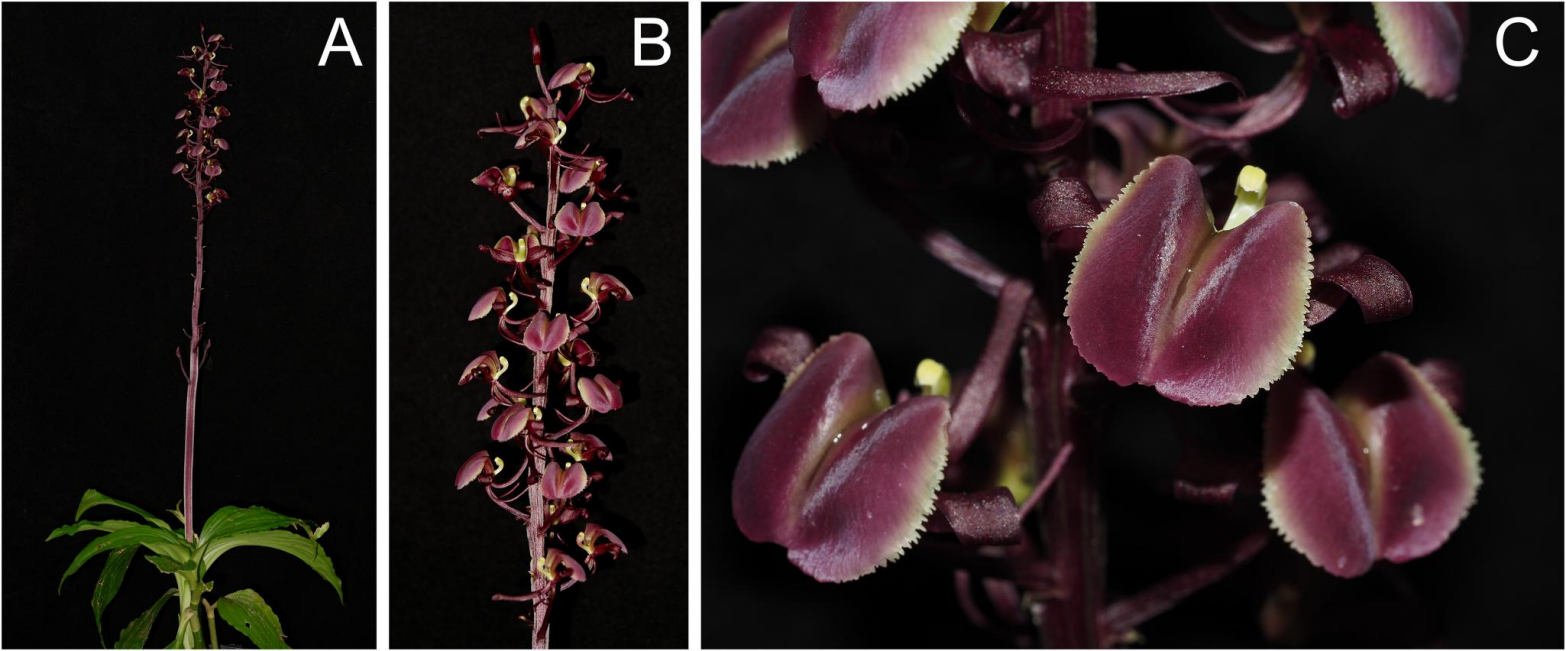
*Liparis gigantea* N2730665 (photos were taken by the author Caixia Peng). (A) The whole plant; (B) inflorescence and flowers; (C) flowers dark purplish red throughout; dorsal sepal 18–20 mm; lip obovate-elliptic with margins conspicuously denticulate.

The living plant of *L. gigantea* (Figure 1) was initially collected in Yunfu City (111°24′ E, 23°13′ N), located in the western Guangdong Province, China, and cultivated in the greenhouse at the Rare and Endangered Plant Conservation Center of the South China National Botanical Garden (SCBG) (Caixia Peng, pengcx@scbg.ac.cn) with the cultivation ID N2730665.

Total genomic DNA was isolated from the young leaf tissue using the Trelief Hi-Pure Plant Genomic DNA Kit (Tsingke Biological Technology Co. Ltd., Beijing, China) following the manufacturer’s instructions. Whole genome sequencing and library construction were performed using the DNBSEQ sequencing platform of Beijing Genomics Institute (BGI), Wuhan, China. Subsequently, the cp genome was *de novo* assembled using the GetOrganelle pipeline (Jin et al. 2020). The PGA (Plastid Genome Annotator) pipeline was selected for the annotation of the cp genome (Qu et al. 2019). Reference genomes of *Liparis loeselii* (MF374688), *Liparis pingtaoi* (MN627758), *Liparis bootanensis* (MN627759), *Liparis nervosa* (MN641753), and *Liparis makinoana* (MN686020) were downloaded from the NCBI GenBank database. Manual inspections and corrections were performed with Geneious Prime 2023 (https://www.geneious.com). The cp genome map was generated by CPGView (Liu et al. 2023).

Maximum-likelihood (ML) phylogenetic tree was constructed based on the cp genome sequence data of *L. gigantea*, as well as those of 13 other species of tribe Malaxideae obtained from the NCBI database. Following the previous study, *Cephalanthera longibracteata* and *Epipactis purpurata* were selected as outgroups (Chase et al. 2015; Yang et al. 2023). All 16 sequences were aligned using the MAFFT Alignment plug-in of Geneious Prime 2023 (https://www.geneious.com). IQ-Tree 1.6.12 (Nguyen et al. 2014) was used to create the phylogenetic tree under the best-fit model TVM+F + R2 (Kalyaanamoorthy et al. 2017) with 1000 SH-aLRT tests and 1000 ultrafast tests bootstrap approach (UFboot) (Guindon et al. 2010; Minh et al. 2013). The phylogenetic tree was visualized by iTOL v6.9 (Letunic and Bork 2024).

## Results

**Figure 2.**
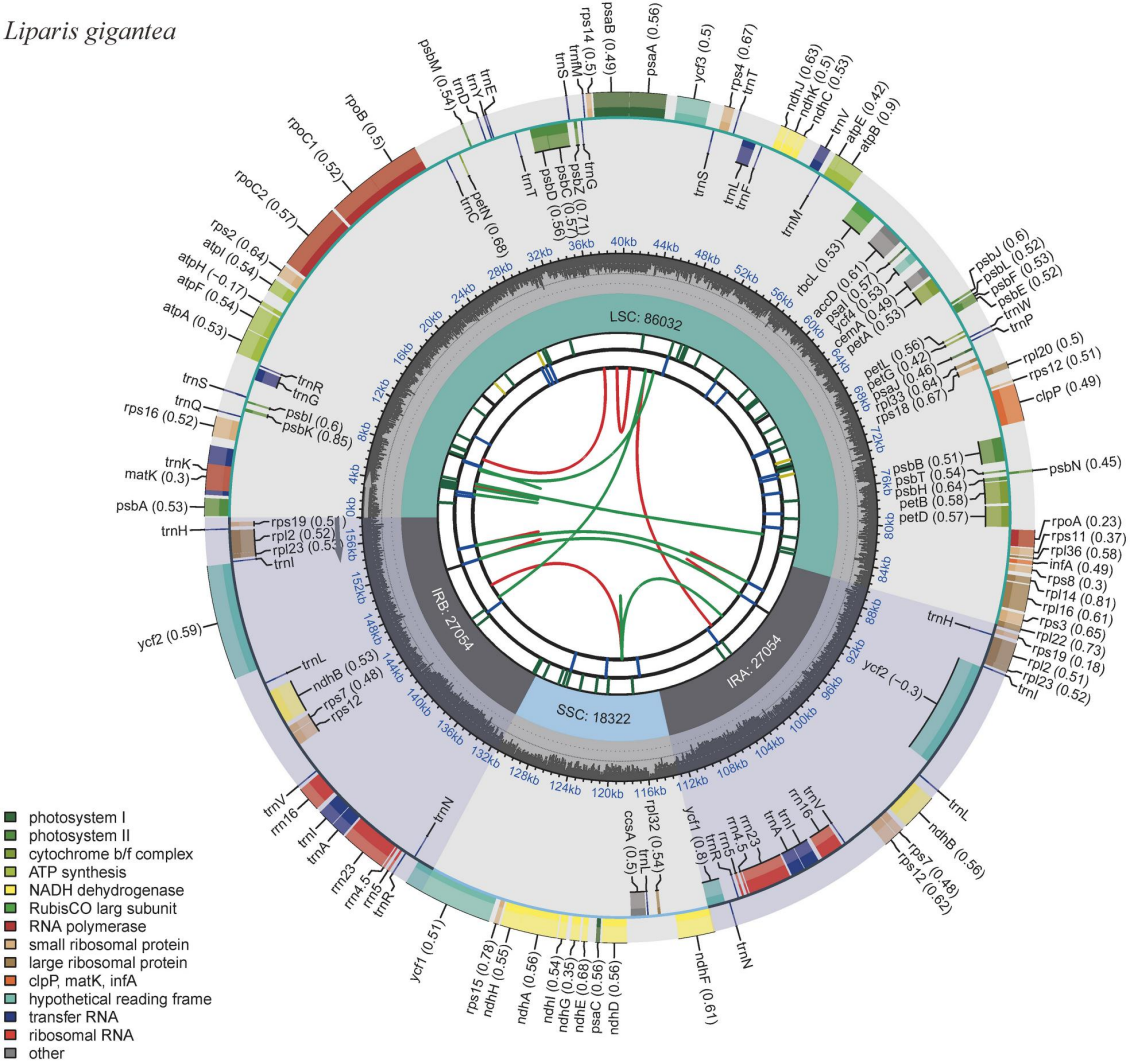
The complete chloroplast genome map of *Liparis gigantea*. From the center outward, the first track shows the dispersed repeats, and the second track shows the long tandem repeats, the third track shows the short tandem repeats or microsatellite sequences. The LSC, SSC, IRA, and IRB are shown in the fourth track. The GC content is plotted on the fifth track. The genes are shown on the sixth track.

3.33 GB sequencing data of *L. gigantea* was generated with whole genome sequencing, and 1.20 × 10^7^ clean reads were obtained after data filtering. The cp genome of *L. gigantea* is 158,462 bp in length with a typical quadripartite structure (Figure 2) and the mean coverage depth is 733.2 (Figure S1). The large single-copy region (LSC) is 86,032 bp in length; the small single-copy region (SSC) is 18,322 bp in length. These two regions are separated by two inverted repeat regions (IRA and IRB) of 27,054 bp each. The overall GC content of the cp genome is 36.9%. It contains 133 genes, including 87 protein-coding genes, 38 tRNA genes, and 8 rRNA genes. Within the CDS, 13 genes exhibit cis-splicing, with two genes containing two introns (*ycf3* and *clp*P), and 11 genes harboring a single intron, including two copies of *rpl2* and two copies of *ndh*B (Figure S2). The structure of the trans-splicing gene *rps12* has also been elucidated (Figure S3).

**Figure 3.**
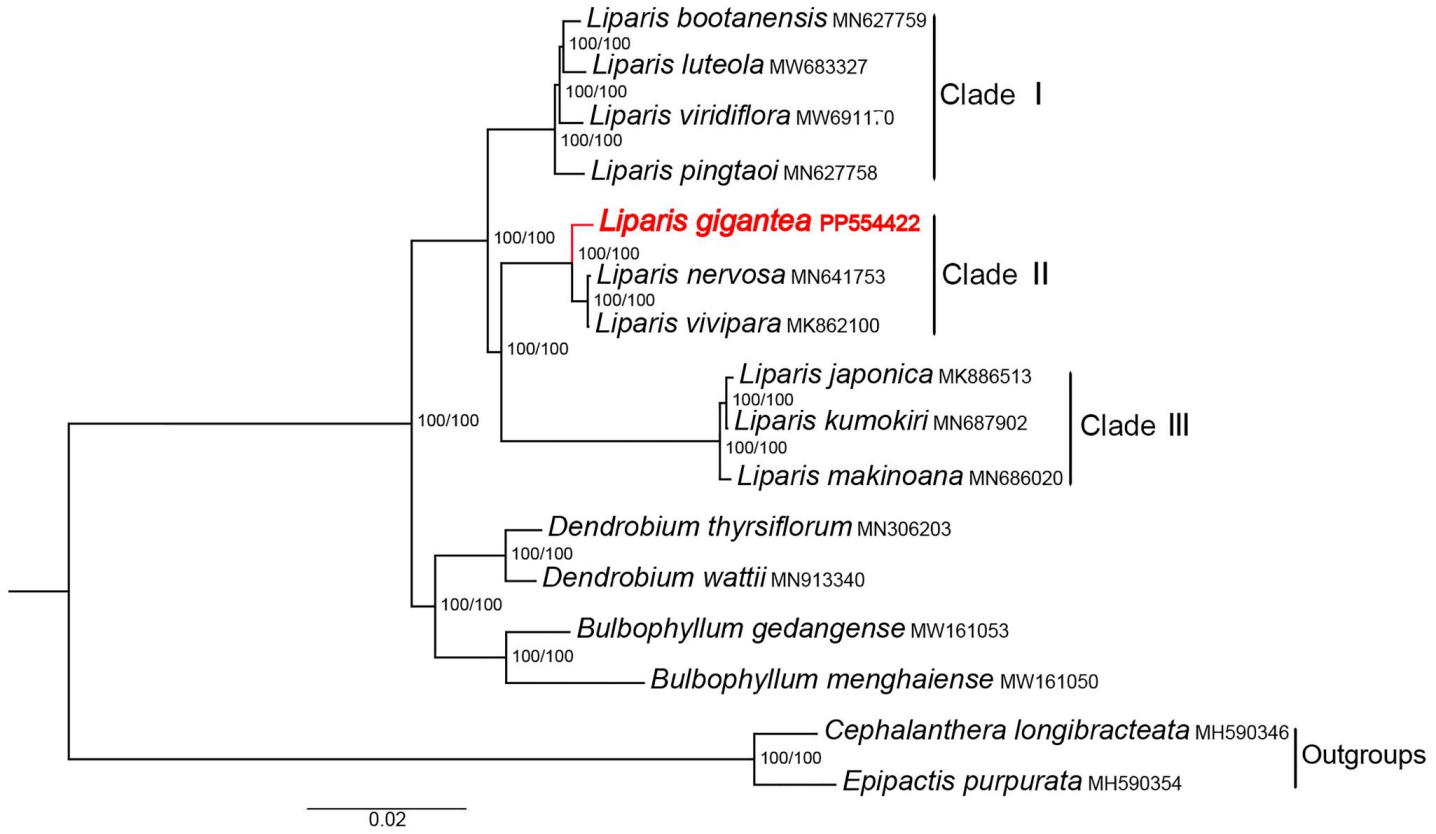
The maximum-likelihood (ML) phylogenetic tree of *Liparis* and its related genera in tribe Malaxideae based on 16 complete chloroplast genomes, showing the position of *Liparis gigantea* (in red). Numbers at nodes represent SH-aLRT support values (left) and UFboot support values (right), respectively. The following sequences were used: *L. bootanensis* MN627759, *L. luteola* MW683327, *L. viridiflora* MW691170, *L. pingtaoi* MN627758, *L. nervosa* MN641753 (Zhang et al. 2020), *L. vivipara* MK862100 (Zhang et al. 2019), *L. japonica* MK886513, *L. kumokiri* MN687902, *L. makinoana* MN686020, *Dendrobium thyrsiflorum* MN306203, *D. wattii* MN913340, *Bulbophyllum gedangense* MW161053 (Tang et al. 2021), *Bulbophyllum menghaiense* MW161050 (Tang et al. 2021), *Cephalanthera longibracteata* MH590346 (Lallemand et al. 2019), and *Epipactis purpurata* MH590354 (Lallemand et al. 2019).

SH-aLRT support values and UFboot support values of each node are both 100, indicating that the ML tree has a high level of reliability. The phylogeny recovered *L. gigantea* as the sister lineage to the clade consisting of *L. nervosa* and *L. vivipara* (Figure 3).

## Discussion and conclusion

The ML phylogenetic tree shows that *Liparis* s.l. can be divided into three clades (Figure 3). Clade I is composed of epiphytes. Taxa in clade II and clade III are terrestrial plants. Clade II consists of terrestrial *Liparis* species with plicate leaves, including *L. gigantea*. Clade III comprises terrestrial *Liparis* species with conduplicate leaves. Our results based on cp genome sequence data are consistent with previous studies using sequence data from nuclear ITS and chloroplast *mat*K regions (Cameron 2005; Li and Yan 2013; Tang et al. 2015; Li et al. 2020; Kumar et al. 2022).

The findings of our study indicate that the terrestrial *Liparis* species with similar plicate leaves form a monophyletic group, clearly separate from taxa with conduplicate leaves. The complete cp genome of *L. gigantea* reported in this study will enrich the cp genome resources and facilitate further research and utilization of this medicinal plant.

## Supplementary Material

Supplementary_Materials.docx

## Data Availability

The complete cp genome sequence generated in this study was submitted to the NCBI database (https://www.ncbi.nlm.nih.gov/) with GenBank accession number: PP554422. The associated BioProject, SRA, and Bio-Sample accession numbers are PRJNA1103398, SRR28778994, and SAMN41054089, respectively.
