# Supplementary material for: Characterization of the complete chloroplast genome of Liparis gigantea (Orchidaceae)
Source: Mitochondrial DNA B Resour. 2024 Oct 16;9(10):1409–13. doi: 10.1080/23802359.2024.2415134 (PMC11486071; doi:10.1080/23802359.2024.2415134)
Supplement: Supplementary_Materials.docx [file TMDN_A_2415134_SM0537.docx]

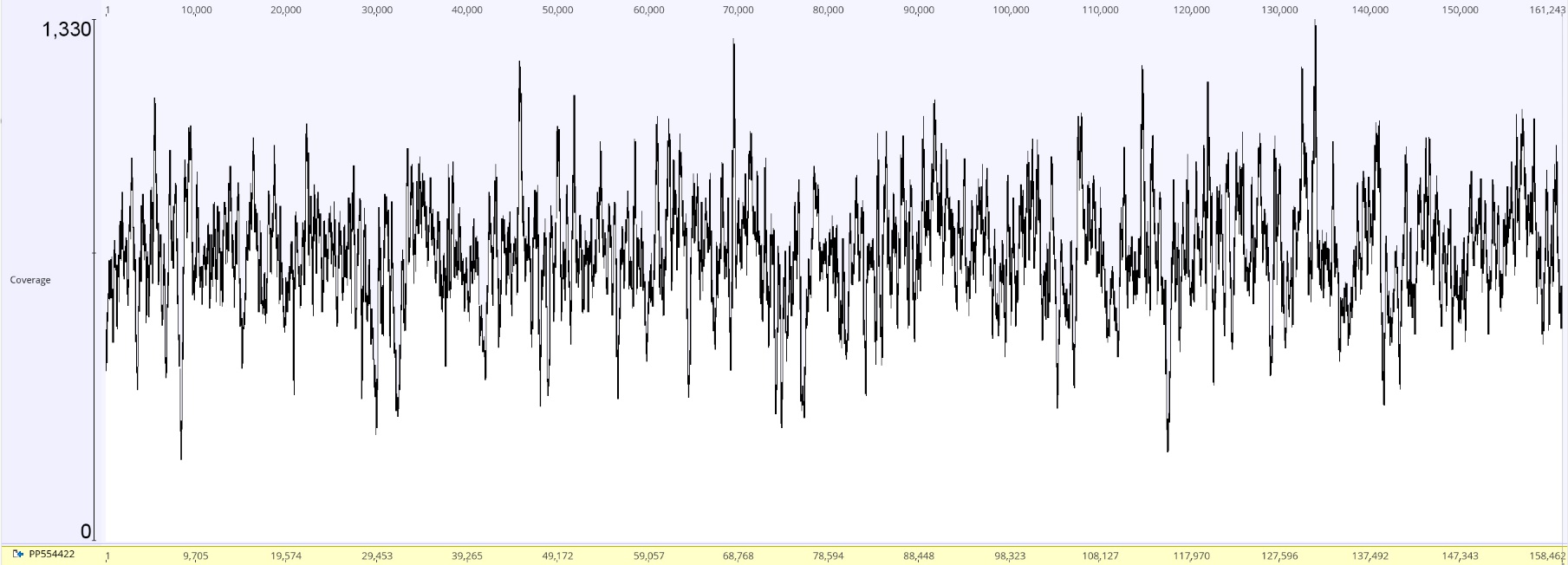


Figure S1. The coverage depth of the chloroplast genome of *Liparis gigantea*. The mean coverage depth of the chloroplast genome is 733.2, the minimum coverage depth is 207, and the maximum coverage depth is 1330.


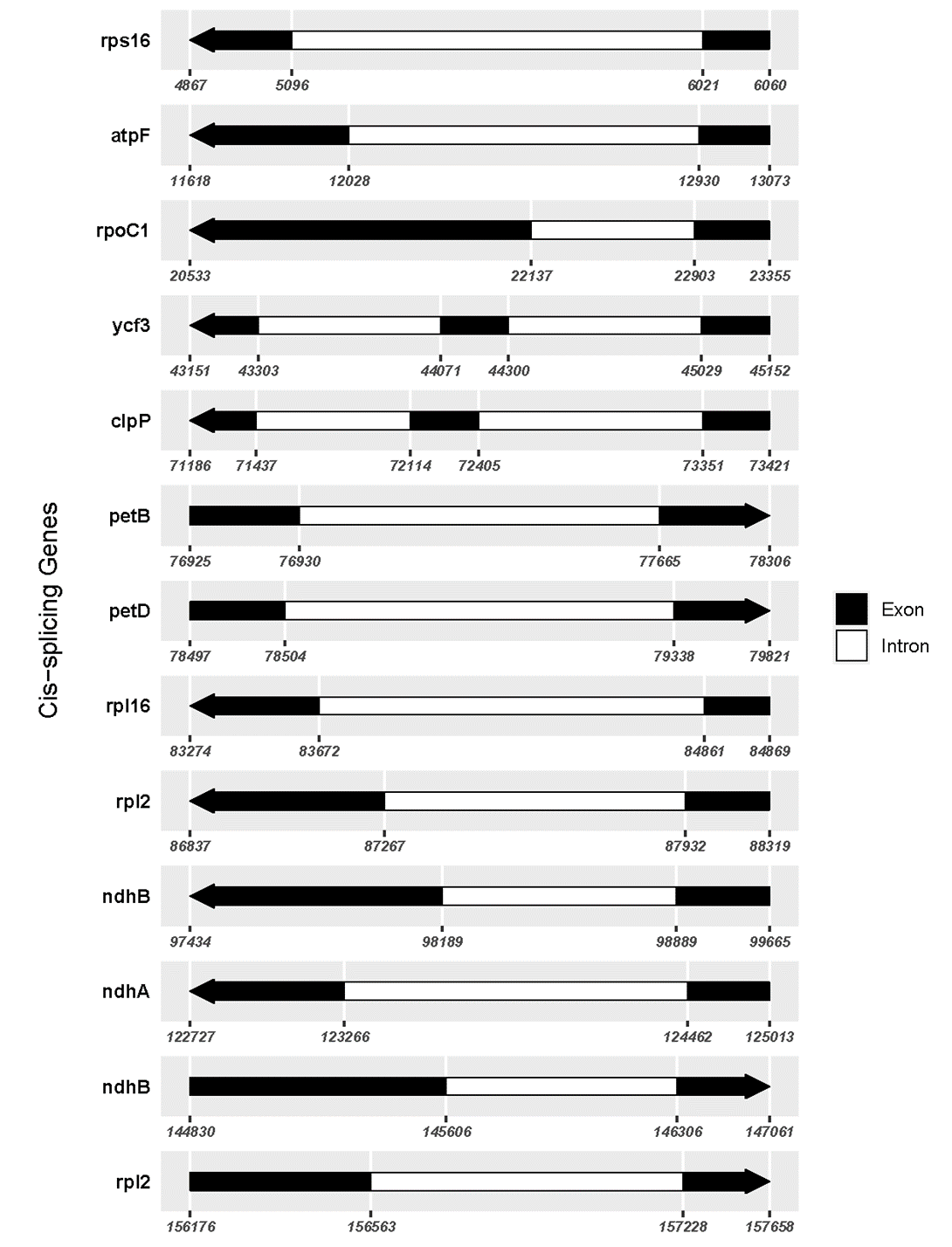


Figure S2. Schematic map of the cis-splicing genes in *Liparis gigantea* chloroplast genome.


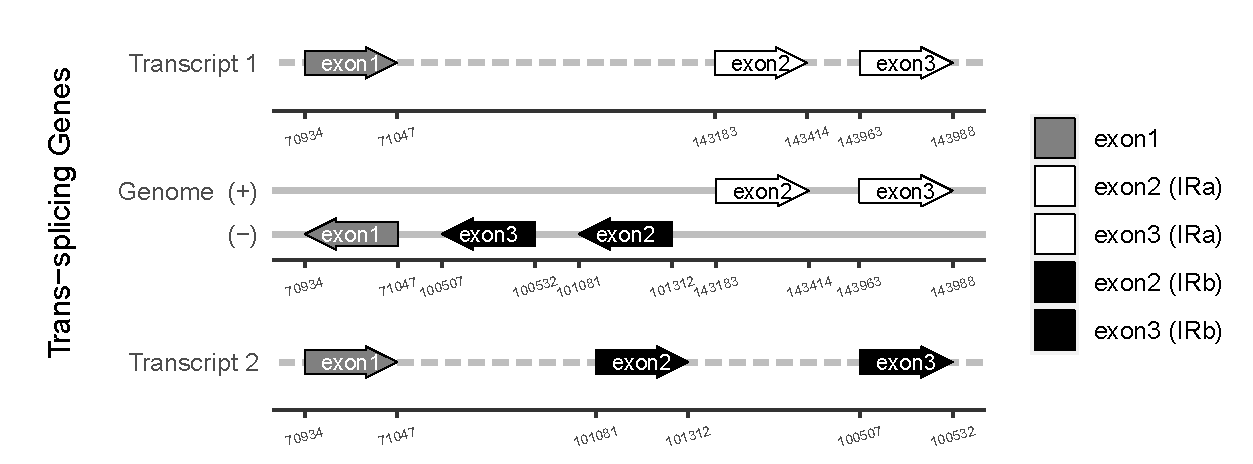
Figure S3. Schematic map of the trans-splicing genes in *Liparis gigantea* chloroplast genome.
